# Medication Nonadherence Among US Immigrants: Complex Roles of Insurance, Citizenship, and Length of Time in the United States

**DOI:** 10.3390/healthcare14142201

**Published:** 2026-07-21

**Authors:** Thomas D. Graves, Wei-Chen Lee, Daniel P. Leblanc, Kaitlin R. Leckie

**Affiliations:** 1 John Sealy School of Medicine, University of Texas Medical Branch, 301 University Blvd., Galveston, TX 77555, USA; thgraves@utmb.edu (T.D.G.); dpleblan@utmb.edu (D.P.L.); 2 Department of Family Medicine, University of Texas Medical Branch, 301 University Blvd., Galveston, TX 77555, USA; kaleckie@utmb.edu

**Keywords:** medication adherence, medication, immigrant, public health

## Abstract

**Objective**: Medication accessibility and adherence are essential factors in healthcare as they ensure patients can follow their doctor’s guidance, enhance treatment success, and lower healthcare costs. Yet, immigrants—a population facing structural disadvantages—remain underrepresented in adherence research. Drawing upon the assimilation theory, this study aims to identify independent and combined effects of cost-related medication nonadherence among immigrants. **Study design**: This is a secondary data analysis using the 2019–2022 National Health Interview Survey. The data included individuals who self-reported that they were born outside of the U.S. and were aged 18 years and older. **Methods**: Over four years, the total weighted sample is 53,739,380, and 52.7% had taken any medication within 12 months before the survey. Chi-squared tests were conducted to examine the differences in characteristics between people currently with and without medications and whether insurance, citizenship, and time in the U.S. affected their medication accessibility and adherence. Any *p*-value less than 0.05 is considered statistically significant. **Results**: Compared to immigrants without medications, immigrants with medications were more likely to have U.S. citizenship (53.4% > 36.8%, *p* < 0.001). Those who entered the U.S. less than five years ago are more likely to delay getting medical care due to cost (15.1% > 8.5%, *p* = 0.0022). **Conclusions**: Certain aspects of healthcare become more accessible for those living in the U.S. for more than five years. There are various possible explanations for these findings, including the 5-year policy required for permanent residents to access Medicaid. Insurance alone does not eliminate cost-related barriers. Targeted policy interventions for recently arrived and uninsured immigrants are needed to close these structural gaps.

## 1. Introduction

Medication adherence is essential in healthcare as it ensures that patients can carry out the plans recommended by their doctors. Sixty-six percent of the United States population took prescription medications in 2021 [1]. Nonadherence to recommended treatments can contribute to frustration felt by patients and providers, increase avoidable deaths, and increase healthcare costs [2]. Without adherence, medications are not able to work to the best of their ability and can worsen many chronic diseases, including hypertension, hyperlipidemia, and diabetes [3]. Additionally, proper adherence to medications is critical in reducing acute emergencies, such as stroke and other vascular emergencies [4]. Therefore, a primary emphasis should be placed on making sure patients can start and, more importantly, continue using their medication for the proper amount of time.

Numerous studies have been published analyzing cost-related medication nonadherence using the National Health Interview Survey among various age groups, disease status groups, and ethnicities but not necessarily in the migration context [5,6,7]. Making up 13.7% of the population, encompassing over 45.3 million people, immigrants are a significant portion that cannot be ignored [8]. Yet, immigrants are historically underrepresented in healthcare research, which can lead to policy decisions and healthcare studies that are less applicable and beneficial to immigrant populations [9]. A review of CDC-supported surveillance systems found that, while 80% collected data on ethnicity, only 32% collected data on place of birth [10]. Additionally, immigrants are already among the most vulnerable populations in the United States due to a general lack of access to food, housing, and transportation and a commonly lower socioeconomic status [11]. It is no surprise that a lack of access to medications compounds these difficulties and creates a complex array of hardships that interfere with their life in the United States. In addition to acclimating to cultures outside of their own and often speaking a different language, immigrants often face financial barriers in finding stable jobs with benefits that allow them to maintain a healthy lifestyle. Immigrants are often forced to work in poorer conditions with less stability, making a solid financial base harder to achieve [12]. Without financial security, stress and hardship become more difficult, leading to worse health and safety outcomes.

This study aims to identify reasons for cost-related medication nonadherence among immigrants. The 2019 National Pharmaceutical Council has offered a framework of medication access in which it identified affordability as a central element of access [13]. However, it did not include immigration-related barriers, such as citizenship and time in the U.S. Insurance status, citizenship, and time spent in the United States are explored to highlight differences in immigrant populations related to adherence, compliance, and financial barriers that prevent them from receiving the same healthcare as their U.S.-born counterparts [14,15]. Whether a person decided to leave the immigrant culture behind (level of assimilation) and the economic opportunities this immigrant receives are also tied to how likely they will be susceptible to downward immigration and becoming marginalized [16]. These variables were chosen because they are commonly cited reasons for hardship in immigrant populations in the United States. Immigrants who are not citizens have less access to insurance, which is related to worse health outcomes [17]. Additionally, recent immigrants have less access to insurance than those who have been in the country for longer periods of time [15]. Even with both insurance and citizenship, many immigrants remain fearful of accessing assistance programs due to cultural differences [18]. In short, investigating the independent and combined effects of insurance, citizenship, and time in the U.S. is still needed.

This study aims to explore the factors involved in immigrants’ cost-related medication nonadherence to guide interventions in public policy to improve their health outcomes and to better represent the immigrant population in healthcare literature. The primary outcomes were self-reported delayed/forgone medical care, medication, and mental health services. With a better understanding of which groups of immigrants have increased difficulty adhering to their healthcare, policy interventions can be better specialized to target higher-risk groups, such as new immigrants or those lacking insurance. The findings may also inform how resources can be prioritized in forming outreach programs and community health programs in areas with higher-risk immigrant populations to help improve accessibility and adherence to healthcare and prevent significant cost burden on individuals and health systems.

## 2. Materials and Methods

### 2.1. Date Source

The data for this study were drawn from the 2019–2022 National Health Interview Survey (NHIS), which collects a broad range of data on health topics through personal household interviews [19]. Every year, field staff conduct more than 30,000 confidential interviews. The NHIS sample is stratified by state and represents the entire nation’s noninstitutionalized individuals or dwelling units. Due to the questionnaire redesign in 2019, our study only used the data from 2019 and onwards to reach the maximum sample size [20].

### 2.2. Study Sample

NHIS has three major parts: roster section, sample adult questionnaire, and sample child questionnaire [21]. Individuals who self-reported that they were born outside of the U.S. and were aged 18 years and older are eligible for this study. The adult interview data provides detailed information on health status and demographics, such as employment and marital status. From 2019 to 2022, the total unweighted adult sample is 110,001, including 89,404 (81.28%) who self-reported that they were born in the U.S. and 20,597 (19.72%) who self-reported that they were not born in the U.S. or were not sure about their birthplace. Weighted sample sizes across the four years were similar at 13,096,129, 13,216,249, 13,278,122, and 14,148,880 for years 2019, 2020, 2021, and 2022, respectively. Among 20,597 who might not be born in the U.S., about 11,451 adults (54.6%) indicated that they took medications at any time in the past 12 months before the survey, and 9146 (44.4%) did not.

### 2.3. Measurements

**Dependent Variables:** The NHIS data regularly monitors delayed or unmet need for medical care due to cost as well as non-financial barriers to care (e.g., transportation or busy work schedule to make an appointment) [21]. Our study was focused on the financial barrier encountered by immigrants. The primary outcomes are centered on responses to ten questions to examine whether a person has had any financial barriers to accessing healthcare and medications in the past 12 months before the survey (except for Question 2). The key questions are (1) having problems paying or unable to pay medical bills, (2) currently unable to pay medical bills, (3) medical care delayed due to cost, (4) delayed filling prescription to save money, (5) delayed getting mental health counseling or therapy due to cost, (6) needed but could not afford medical care, (7) needed but could not afford prescription medicines, (8) needed but could not afford mental health, (9) took less medication to save money, and (10) skipped medication doses to save money. Responses are classified into two categories: 1 = Yes (nonadherence) and 0 = No (adherence).

**Independent Variables:** The primary independent variable is whether a survey respondent took medicines in the past year before the survey (1 = Yes and 0 = No). The secondary independent variables are their insurance status, citizenship status, and number of years in the U.S. Individuals reported what kinds of health insurance they have and whether their health insurance was offered to them through their workplace. Then, a new variable was generated with three categories (0 = Uninsured, 1 = Insured through Other Sources, 2 = Insured through Workplace). Regardless of their birthplace, everyone was also asked whether they are a citizen of the United States, and their responses are classified into two categories (1 = Yes and 0 = No). Finally, four new variables were generated to see the combined effects of disadvantaged factors, including group 1 (who are uninsured and do not have citizenship), group 2 (who are uninsured and have been in the U.S. for less than 5 years), group 3 (who do not have citizenship and have been in the U.S. for less than 5 years), and group 4 (with all three disadvantaged factors: uninsured, do not have citizenship, and have been in the U.S. for less than 5 years).

**Covariates and Sampling Weights:** Multiple studies have explored the relationship between demographics (e.g., age) and financial barriers (e.g., education and employment) using NHIS data [7,22,23,24]. Our study has chosen key influential factors (insurance, citizenship, and time in the U.S.) as the predictors. Other demographic factors such as gender and age were not discussed in our study because the composition of immigrant population varies each year. Education, employment, and insurance are highly correlated, and hence, our study was focused on insurance only. The survey year before (2019) and after (2020–2022) the pandemic shows a statistically significant difference for the first question (problem with paying) after we used Bonferroni correction for multiple comparisons and set 0.005 (=0.05/10) as the *p*-value (Table A1). Therefore, “year” was adjusted in the multivariable model for Q1. Our additional analyses, including various covariates (e.g., age, gender, and education level), did not find differential findings, where three key factors (insurance, citizenship, and time in the U.S.) remain significant associations with our cot-related adherence outcomes.

Next, this study used four yearly NHIS datasets from 2019 to 2022. To ensure that survey estimates are representative of the U.S. noninstitutionalized individuals, the NHIS employs weighting procedures to account for the complex sampling design and nonresponses [25]. The STATA commands for generating pooled weights are as below:

drop if age > 17 & partweight = 0 & year = 2020(1)

gen pooled_weight = sampweight(2)

replace pooled_weight = partweight if year = 2020 & age > 17 & age != .(3)

gen pooled_weight_adj = pooled_weight/4(4)

**Statistical Analysis:** Descriptive analysis along with chi-squared tests were conducted to examine the differences in characteristics between people with and without medications. Bivariate analyses were performed to examine whether insurance, citizenship, and time in the U.S. affected their healthcare accessibility and medication adherence among those who self-reported having prescriptions in the past 12 months. Focusing on those with prescriptions was the key to knowing what factors influenced their nonadherence. As each individual may experience one or more than one disadvantage in health, we investigated both independent and combined effects of three factors generating empirical evidence on what motivated immigrants to delay or forgo care due to costs. Logistic regression analyses were further conducted to examine combined effects (odds ratio and 95% CI) of one or more disadvantaged factors on ten respective financial barriers (1 = yes, delaying or forgoing the care, 0 = no). Using citizenship as the parameter, the effect size ranged from 0.079 for Q1 to 0.0874 for Q10. The higher Cohen’s d indicates a greater level of nonadherence due to no citizenship. All analyses were done using the STATA v18.0 software package [25], and a two-sided *p*-value less than 0.05 was considered statistically significant. Claude ai was used to create the forest chart showing odds ratios for 10 different questions. Sensitivity analysis was conducted to see if the current use of any medication might be related to an individual’s financial hardship and medication nonadherence among adults who self-reported they did not have prescriptions in the past 12 months (Table A2).

## 3. Results

### 3.1. Subject Background

Table 1 presents demographic data of the foreign-born adults surveyed in the 2019–2022 National Health Interview, with columns representing those who did and did not receive medication in the last 12 months. Citizens and immigrants who had been in the U.S. for 15+ years, as well as those with insurance, were more likely to have received medication (*p* < 0.001).

### 3.2. Single Effect

Table 2 examines medication accessibility and adherence among those who need medication in the survey cohort in the context of insurance status, citizenship status, and time in the United States. Choosing people with prescriptions was critical to know what factors influenced their cost-related nonadherence despite their needs for healthcare. There are significant differences in the use of medications between those who have insurance and those who do not have insurance. Notably, uninsured individuals reported much higher rates of financial barriers, with 36% of uninsured individuals delaying medical care due to cost, compared with only 5.9% and 7.1% of insured individuals delaying (*p* < 0.001) (Question 3). Additionally, 33.9% of the uninsured population reported needing medical care but were unable to afford it, compared with 5.6% and 7.2% of insured individuals (*p* < 0.001). Non-citizens reported significantly more difficulty than citizens across all questions, indicating financial, access, and adherence barriers (excluding Question 5, which related to delaying mental healthcare due to cost) (*p* < 0.001).

This trend of experiencing barriers to care was consistent among immigrants who had been in the U.S. less than 5 years compared with those who had been in the U.S. 5–15 years or more than 15 years (Question 3, 15.1% vs. 8.5% vs. 8.9%, *p* = 0.0022) (Question 6, 14.6%, 9.2%, 8.2%, *p* = 0.0019). We further compared the nonadherence rate of the two groups by immigration time and found that more recent immigrants (<5 years) had significantly higher odds than long-term immigrants (15+) in Question 2 and higher odds than both mid- and long-term immigrants in Questions 3, 5, and 6. Of note, a separate analysis was conducted to see if immigrants without medication have financial barriers (see Table A1). Our supplement shows the same pattern: immigrants who are uninsured, lack citizenship, and have been in the U.S. for a shorter time are more likely to be unable to get health services, implying that there is no selection bias in our approach. The post hoc comparisons using Bonferroni correction showed that three groups were significantly different in their levels of nonadherence (*p* < 0.001).

### 3.3. Combined Effect

Table 3 analyzes the difficulties faced by the survey cohort focusing on insurance status and immigrant time status as compounding risk factors. Notably, being uninsured and a non-citizen increased the odds of financial barriers (delaying or forgoing the care) significantly, with the highest risk being 8.02 times higher than individuals with insurance and citizenship (95% CI: 6.46–9.96, *p* < 0.001). The uninsured and non-citizen category reported significant barriers to care across all categories (*p* < 0.001). Other combinations of categories, including being a short-term immigrant, reported similar significant difficulties affording medical care and delaying care due to cost (*p* < 0.001). However, these categories reported less significant difficulty in other dimensions of barriers to care, such as delaying filling prescriptions, taking less medication to save money, or skipping medication to save money (Questions 4, 9, 10).

**Figure 1:**
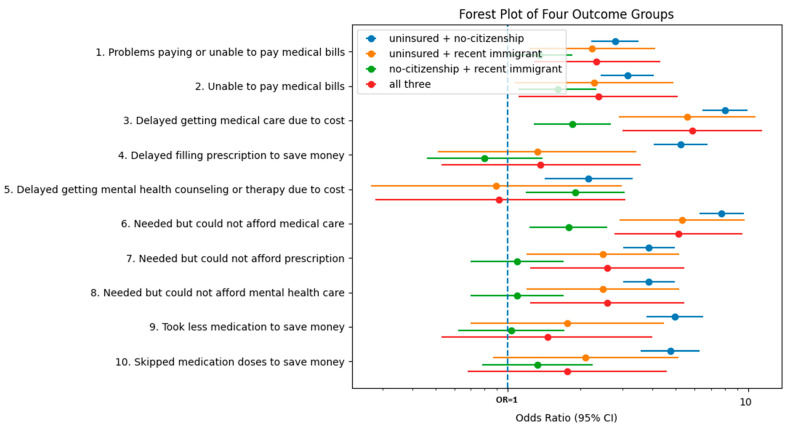
Comparing odds ratios among four groups across 10 questions.

Figure 1 illustrates the differences in odds ratios among four combined groups across 10 survey questions. Of all four combined groups, the “uninsured + no citizenship” consistently had higher odds of delaying or forgoing healthcare, whereas the “no citizenship and recent immigrant” had lower odds. Next, self-reported medication nonadherence was highly related to decisions to delay medical care due to cost (OR = 8.02), followed by unable to afford medical care (OR = 7.77).

## 4. Discussion

The 2019 National Pharmaceutical Council has offered a framework of medication access in which it identified affordability as a central element of access [13]. Meanwhile, segmented assimilation by Portes and Zhou (1993) provides additional insights into the association between economic opportunities and immigrants’ outcomes [16]. This study aligned with both frameworks and leveraged the strengths of NHIS to investigate cost-related medication nonadherence and focus on combined effects of insurance (availability of any health plans), citizenship (eligibility for any social benefits), and length in the U.S. (familiarity of U.S. health systems) among immigrants. Similar to the literature, insurance coverage was the largest disparity driver among immigrants, followed by citizenship and time in the U.S. [6,26,27]. Multiple financial barriers dropped significantly when immigrants received insurance or citizenship, but only four of 10 areas dropped by a longer time in the U.S., including unable to pay medical bills (*p* = 0.0205), delayed getting medical care (*p* = 0.0022), delayed getting mental health services (*p* = 0.0373), and needed but could not afford medical care (*p* = 0.0019). The finding indicates that a shorter time in the U.S. was a hurdle for medical and mental care, but time in the U.S. would not change their medicine taking behaviors. While these findings demonstrate an interesting dynamic among insurance, citizenship, and time in the U.S., the findings also inform more research to investigate specific barriers to medical care, mental health services, and medication.

### 4.1. Main Findings

Our study found uninsured immigrants and non-citizens were significantly less likely to have prescription medications than insured immigrants and citizens, which reflects cumulative disadvantages leading to amplified barriers to adherence. We also found that immigrants with prescription medications are significantly more likely to have lived in the United States for 15 or more years. This may emphasize the importance of integration into the United States, whether through time spent in the country or the result of obtaining citizenship or insurance. Another study exploring cost-related nonadherence found that citizens were more likely than non-citizens to have prescription medications [28]. Accordingly, a significant focus on immigrant public health policy should be on working with these populations to help them obtain citizenship, employment, or services that afford better access to insurance and health-supportive resources. Even though, with time, more immigrants can get prescription medications, immigrants may be more likely to need these prescriptions the longer they have been in the United States if they cannot obtain congruence with cultural norms of stability [29]. Therefore, a greater emphasis on preventive services and resources for recent immigrants is needed because uncontrolled diseases such as hypertension can lead to increased risk of cardiovascular disease and death [30].

### 4.2. Exclusion of Other Factors

The literature using health conditions such as asthma and diabetes has found that insurance or comorbidities could drive an increased risk of cost-related medication nonadherence (CRMN) [7,22]. However, none of these studies explore the interactions between immigration, insurance, citizenship, and length in the U.S. in depth. Another interesting finding of ours is whether the study participants self-reported they used medications in the past 12 months or not; the CRMN patterns are similar. Additionally, our study illustrates that people with current medication use were more likely to report CRMN than people without. For example, 29.3% of people using medications reported problems with paying medical bills due to insurance (Table 2), but the corresponding number is only 13.6% among people who recently did not use medications (Table A2). In other words, people who were using medications were more vulnerable to access medical care, mental health counseling, and medications when the cost was high. The finding underscores their unmet needs due to costs, and strategies to close the gap are imperative.

### 4.3. Domestic Policy Implications

The data presented suggest that a more adaptive approach is needed from the U.S. healthcare system to better meet the needs of an evolving population. Although an initial interpretation of the data may suggest that offering insurance to all immigrants would be beneficial, it would do nothing to address the accompanying barriers, such as education status and employment impact on the ability to access and afford care. Therefore, multiple levels of intervention will be important to address these barriers. It has been shown that, when health systems partner with social systems, they can mitigate difficulties that are faced by vulnerable populations [31]. Additionally, approaches and services that improve cultural competence and health literacy have been shown to improve health outcomes [32,33]. Navigating the current healthcare system in the United States is nuanced and challenging even for those with adequate insurance, financial means, and minimal barriers to care [34]. For immigrants who face many challenges shown in the data above, this task is much more difficult. For the healthcare system to better address these needs, changes will need to be made in the breadth of insurance coverage available, the cultural competence of the care provided, emphasis on early preventive care, and improvements in research to guide care [35]. Longitudinal studies would be of great benefit to track the health outcomes of immigrants over time, with close attention to insurance access, citizenship status, and amount of time spent in the United States. It should be recognized that these studies present challenges to being executed effectively, such as loss to follow-up [36]. To better understand the health needs and vulnerabilities of immigrants, data determining if access to and usage of medication change upon receiving insurance, gaining citizenship, and spending more time in the United States would also be useful.

### 4.4. International Comparisons

The findings of this study are not only relevant to the United States. Causes of underrepresentation of immigrants in public health research are multifactorial and seen in other countries, such as the United Kingdom, where a study found that many immigrants were less likely to respond to surveys due to mistrust of the government or inability to read the survey material in their native language [37]. Professional translation of survey materials is important, as a study in Australia found that 24% of migrants found sexual health surveys difficult to understand [38]. Because of this, both in the United States and abroad, it is necessary to use proper translation services with sufficient attention to immigrant languages to ensure that they are able to complete surveys and be considered in the data that guide policy making. This process involves multiple independent translators, extensive review, and pretesting committees to ensure translated material is understood in the target language [39]. Other methods for increasing immigrant participation in public health research to better meet their needs for policy decision making include partnering with immigrants in the design of studies to better suit the needs of target populations. A review of 161 public health research studies found that community-based participatory research, which involves engaging immigrants in the design and implementation of surveys of the target population, was effective in enhancing the research rigor [40]. In order to address the cos-related unmet needs seen in this study, it is necessary to conduct studies in a way that targets underrepresented populations that are at risk of being overlooked.

### 4.5. Study Limitations

This study has several limitations that should be considered. Because these results were gathered from the 2019–2022 National Health Interview Survey, the COVID-19 pandemic likely significantly impacted the results measured. Additionally, covariates such as region of residence were not accounted for, ignoring the potential variability in access to medications and prices of medications in different locations. Due to anti-immigrant portrayal in news and policy, immigrants may be less likely to participate in surveys due to the fear that they be identified and targeted because of their immigrant status [41]. Immigrants who feel especially at risk of discovery or identification may not be represented in these data, thus failing to include a particularly vulnerable population. Our additional analysis of comparing adherence by three factors did not show any difference between two time periods (2019 vs. 2020–2022) or due to adding confounders like age and sex. However, a more extensive study over a longer period could be performed to see if these results are consistent.

## 5. Conclusions

This study demonstrates that the absence of insurance, citizenship, and familiarity with the U.S. healthcare system independently affected medical nonadherence and that co-occurrence of uninsured and non-citizens substantially amplified the risk. Seeing a doctor, being diagnosed, and having a prescription written are not enough to ensure the health and safety of immigrant populations if they are not able to consistently access these medications over time. This study emphasizes the compounding nature of these variables in limiting immigrant medication adherence and provides future direction on how to resolve these problems moving forward. Change is necessary at all levels of the healthcare system, from pharmacies to social workers, to ensure that immigrants can receive and access medication on a consistent basis and can obtain the services and resources that prevent the need for medication in the first place. Longitudinal research tracking adherence trajectories as immigrants gain insurance, citizenship, and residency would further clarify causal pathways and inform targeted interventions to address the structural barriers.

## Figures and Tables

**Table 1:** Comparison of demographics of foreign-born adults aged 18+ years who used and did not use medications last year.

Had Medications (Rx) in the Past 12 Months or Not	Without	With Rx	*p*-Value
	N = 25,440,212	N = 28,299,168	
**Insurance**			<0.001
Uninsured	6,210,794 (24.4%)	2,765,295 (9.8%)	
Insured through other source	10,305,727 (40.5%)	16,657,010 (58.9%)	
Insured through workplace	8,923,691 (35.1%)	8,876,863 (31.4%)	
**Citizenship = Yes**	9,369,127 (36.8%)	15,117,095 (53.4%)	<0.001
**# of years in the U.S.**			<0.001
Less than 5 years	2,404,689 (12.1%)	1,228,756 (5.3%)	
5 years to less than 15 years	5,050,409 (25.4%)	3,783,857 (16.3%)	
15 years or more	12,432,350 (62.5%)	18,172,785 (78.4%)	

**Table 2:** Examining causes of medication nonadherence among foreign-born adults who used medications last year (single effect).

Insurance Status	Uninsured	Insured Through Other Source	Insured Through Workplace	*p*-Value
	(N = 2,765,295)	(N = 16,657,010)	(N = 8,876,863)	
1. Problems paying or unable to pay medical bills	29.3%	13.0%	12.2%	<0.001
2. Unable to pay medical bills	21.0%	7.8%	6.8%	<0.001
3. Delayed getting medical care due to cost	36.0%	5.9%	7.1%	<0.001
4. Delayed filling prescription to save money	21.8%	4.9%	5.0%	<0.001
5. Delayed getting mental health counseling or therapy due to cost	5.7%	2.4%	3.8%	<0.001
6. Needed but could not afford medical care	33.9%	5.6%	7.2%	<0.001
7. Needed but could not afford prescription	21.8%	6.6.%	6.1%	<0.001
8. Needed but could not afford mental healthcare	21.8%	6.6%	6.1%	<0.001
9. Took less medication to save money	19.2%	4.2%	4.2%	<0.001
10. Skipped medication doses to save money	16.8%	3.8%	4.2%	<0.001
**Citizenship**	**No**	**Yes**	***p*** **-value**	
	(N = 13,182,073)	(N = 15,117,095)		
1. Problems paying or unable to pay medical bills	17.6%	11.5%	<0.001	
2. Unable to pay medical bills	11.4%	6.5%	<0.001	
3. Delayed getting medical care due to cost	13.0%	5.9%	<0.001	
4. Delayed filling prescription to save money	8.6%	4.8%	<0.001	
5. Delayed getting mental health counseling or therapy due to cost	3.6%	2.9%	0.0738	
6. Needed but could not afford medical care	12.4%	5.8%	<0.001	
7. Needed but could not afford prescription	9.8%	6.2%	<0.001	
8. Needed but could not afford mental healthcare	9.8%	6.2%	<0.001	
9. Took less medication to save money	7.5%	4.1%	<0.001	
10. Skipped medication doses to save money	6.9%	3.7%	<0.001	
**Time in the U.S.**	**Less than 5 years**	**5 years to less than 15 years**	**15 years or more**	***p*** **-value**
	(N = 1,228,756)	(N = 3,783,857)	(N = 18,172,785)	
1. Problems paying or unable to pay medical bills	17.7%	13.8%	13.5%	0.1092
2. Unable to pay medical bills	12.5%	9.1%	7.9%	0.0205
3. Delayed getting medical care due to cost	15.1%	8.5%	8.9%	0.0022
4. Delayed filling prescription to save money	5.5%	6.7%	6.3%	0.8039
5. Delayed getting mental health counseling or therapy due to cost	5.4%	2.7%	3.3%	0.0373
6. Needed but could not afford medical care	14.6%	9.2%	8.2%	0.0019
7. Needed but could not afford prescription	8.1%	8.1%	7.5%	0.8562
8. Needed but could not afford mental healthcare	8.1%	8.1%	7.5%	0.8562
9. Took less medication to save money	6.4%	6.0%	5.5%	0.7329
10. Skipped medication doses to save money	7.2%	5.5%	5.0%	0.3296

**Table 3:** Examining causes of medication nonadherence among foreign-born adults who used medications last year (combined effect).

Nonadherence/Adherence	Uninsured + No-Citizenship (n = 2,302,747)	Uninsured + Recent Immigrant (n = 245,468)	No-Citizenship + Recent Immigrant (n = 1,164,132)	All Three (n = 237,557)
1. Problems paying or unable to pay medical bills	2.80 (2.23, 3.51) ***	2.24 (1.23, 4.10) **	1.36 (1.00, 1.85) *	2.34 (1.28, 4.30) **
2. Unable to pay medical bills	3.15 (2.44, 4.06) ***	2.28 (1.07, 4.89) *	1.61 (1.11, 2.33) *	2.38 (1.11, 5.09) *
3. Delayed getting medical care due to cost	8.02 (6.46, 9.96) ***	5.57 (2.89, 10.77) ***	1.85 (1.28, 2.69) **	5.87 (3.01, 11.45) ***
4. Delayed filling prescription to save money	5.23 (4.05, 6.76) ***	1.33 (0.51, 3.42)	0.80 (0.46, 1.39)	1.37 (0.53, 3.56)
5. Delayed getting mental health counseling or therapy due to cost	2.17 (1.42, 3.32) ***	0.89 (0.27, 2.98)	1.91 (1.19, 3.07) **	0.92 (0.28, 3.09)
6. Needed but could not afford medical care	7.77 (6.28, 9.60) ***	5.31 (2.91, 9.71) ***	1.79 (1.23, 2.60) **	5.14 (2.78, 9.50) ***
7. Needed but could not afford prescription	3.87 (3.02, 4.96) ***	2.49 (1.20, 5.18) *	1.09 (0.70, 1.71)	2.60 (1.24, 5.43) *
8. Needed but could not afford mental healthcare	3.87 (3.02, 4.96) ***	2.49 (1.20, 5.18) *	1.09 (0.70, 1.71)	2.60 (1.24, 5.43) *
9. Took less medication to save money	4.96 (3.79, 6.50) ***	1.77 (0.70, 4.46)	1.03 (0.62, 1.72)	1.46 (0.53, 3.99)
10. Skipped medication doses to save money	4.74 (3.57, 6.28) ***	2.11 (0.87, 5.13)	1.33 (0.78, 2.25)	1.77 (0.68, 4.59)

Note: *: *p* < 0.05, **: *p* < 0.01; ***: *p* < 0.00.

## Data Availability

The data presented in this study are openly available in the National Center for Health Statistics at https://www.cdc.gov/nchs/nhis/index.html (accessed on 29 January 2025).

## Appendix A

**Table A1:** Comparison of 10 cost-related nonadherence outcomes between 2019 (pre-pandemic) and 2020–2022 (post-pandemic) among immigrants who used medications in the past 12 months.

% (95% C.I.)	2019	2020–2022	*p*-Value
1. Problems paying or unable to pay medical bills	16.8 (15.1, 18.5)	13.6 (12.7, 14.6)	0.0008
2. Unable to pay medical bills	10.0 (8.6, 11.5)	8.4 (7.6, 9.3)	0.0497
3. Delayed getting medical care due to cost	11.0 (9.6, 12.6)	8.6 (7.8, 9.5)	0.0053
4. Delayed filling prescription to save money	7.5 (6.4, 8.8)	6.2 (5.6, 7.0)	0.0584
5. Delayed getting mental health counseling or therapy due to cost	3.7 (2.9, 4.7)	3.0 (2.6, 3.5)	0.1403
6. Needed but could not afford medical care	10.5 (9.2, 12.1)	8.3 (7.5, 9.2)	0.0064
7. Needed but could not afford prescription	9.4 (8.0, 11.0)	7.4 (6.7, 8.3)	0.0256
8. Needed but could not afford mental healthcare	3.9 (3.1, 5.0)	3.4 (2.9, 3.8)	0.2638
9. Took less medication to save money	6.6 (5.6, 7.8)	5.3 (4.7, 6.1)	0.0455
10. Skipped medication doses to save money	6.1 (5.1, 7.3)	4.9 (4.3, 5.6)	0.0681

**Table A2:** 2019–2022 National Health Interview Survey: foreign-born adults aged 18+ years who “did not” take medications last year.

Insurance	Uninsured	Insured Through Other Source	Insured Through Workplace	*p*-Value
	(n = 6,210,794)	(n = 10,305,727)	(n = 8,923,691)	
1. Problems paying or unable to pay medical bills	13.6%	6.1%	6.7%	<0.001
2. Unable to pay medical bills	9.1%	3.9%	3.5%	<0.001
3. Delayed getting medical care due to cost	18.7%	3.0%	4.6%	<0.001
4. Delayed filling prescription to save money	0.0%	0.0%	0.0%	.
5. Delayed getting mental health counseling or therapy due to cost	0.7%	0.6%	0.7%	0.0113
6. Needed but could not afford medical care	4.4%	1.2%	1.7%	<0.001
7. Needed but could not afford prescription	1.4%	0.5%	0.5%	<0.001
8. Needed but could not afford mental healthcare	1.4%	0.5%	0.5%	<0.001
9. Took less medication to save money	0.0%	0.0%	0.0%	<0.001
10. Skipped medication doses to save money	0.0%	0.0%	0.0%	<0.001
**Citizenship**		**No**	**Yes**	***p*** **-Value**
		**(n = 16,071,086)**	**(n = 9,369,127)**	
1. Problems paying or unable to pay medical bills		9.8%	5.4%	<0.001
2. Unable to pay medical bills		6.2%	3.1%	<0.001
3. Delayed getting medical care due to cost		9.2%	4.3%	<0.001
4. Delayed filling prescription to save money		0.0%	0.0%	.
5. Delayed getting mental health counseling or therapy due to cost		1.9%	2.0%	0.5927
6. Needed but could not afford medical care		8.7%	4.7%	<0.001
7. Needed but could not afford prescription		2.7%	1.7%	0.006
8. Needed but could not afford mental healthcare		2.7%	1.7%	0.006
9. Took less medication to save money		0.0%	0.0%	.
10. Skipped medication doses to save money		0.0%	0.0%	.
**Time in the U.S.**	**Less than 5 years**	**5 years to less than 15 years**	**15 years or more**	***p*-value**
	**(n = 2,404,689)**	**(n = 5,050,409)**	**(n = 17,985,115)**	
1. Problems paying or unable to pay medical bills	7.8%	7.6%	8.4%	0.7066
2. Unable to pay medical bills	5.2%	4.1%	5.3%	0.3562
3. Delayed getting medical care due to cost	6.5%	8.7%	7.2%	0.1379
4. Delayed filling prescription to save money	0.0%	0.0%	0.0%	.
5. Delayed getting mental health counseling or therapy due to cost	1.9%	1.6%	2.0%	0.6581
6. Needed but could not afford medical care	6.8%	9.0%	6.8%	0.0341
7. Needed but could not afford prescription	2.1%	2.3%	2.4%	0.9021
8. Needed but could not afford mental healthcare	2.1%	2.3%	2.4%	0.9021
9. Took less medication to save money	0.0%	0.0%	0.0%	.
10. Skipped medication doses to save money	0.0%	0.0%	0.0%	.
